# Nucleases in *Bdellovibrio bacteriovorus* contribute towards efficient self-biofilm formation and eradication of preformed prey biofilms

**DOI:** 10.1111/1574-6968.12075

**Published:** 2013-02-08

**Authors:** Carey Lambert, R Elizabeth Sockett

**Affiliations:** Centre for Genetics and Genomics, School of Biology, Queen's Medical Centre, University of NottinghamNottingham, UK

**Keywords:** *Bdellovibrio bacteriovorus*, endonuclease, biofilm formation

## Abstract

*Bdellovibrio bacteriovorus* are predatory bacteria that burrow into prey bacteria and degrade their cell contents, including DNA and RNA, to grow. Their genome encodes diverse nucleases, some with potential export sequences. Transcriptomic analysis determined two candidate-predicted nuclease genes (*bd1244*, *bd1934*) upregulated upon contact with prey, which we hypothesised, may be involved in prey nucleic acid degradation. RT-PCR on total RNA from across the predatory cycle confirmed that the transcription of these genes peaks shortly after prey cell invasion, around the time that prey DNA is being degraded. We deleted *bd1244* and *bd1934* both singly and together and investigated their role in predation of prey cells and biofilms. Surprisingly, we found that the nuclease-mutant strains could still prey upon planktonic bacteria as efficiently as wild type and still degraded the prey genomic DNA. The *Bdellovibrio* nuclease mutants were less efficient at (self-) biofilm formation, and surprisingly, they showed enhanced predatory clearance of preformed prey cell biofilms relative to wild-type *Bdellovibrio*.

## Introduction

*Bdellovibrio bacteriovorus* are predatory bacteria that prey upon a wide range of Gram-negative bacteria, entering their periplasm and growing at the expense of the prey macromolecules. Once established in the periplasm, the *Bdellovibrio* rapidly kills the prey (Rittenberg & Shilo, 1970) and then begins to degrade cytoplasmic macromolecules, such as nucleic acids, in a controlled and stepwise manner requiring careful regulation of the hydrolytic enzymes involved (Matin & Rittenberg, 1972; Hespell *et al*., 1975). As replicating *Bdellovibrio* must generate an average of 3–5 genomes of 3.8 Mb from preying upon one cell of *Escherichia coli* with a single genome of similar size, efficient degradation and recycling of prey nucleic acids are predicted to be a pressing issue (in addition to *de novo* synthesis). Analysis of the genome of *B. bacteriovorus* HD100 (Rendulic *et al*., 2004) reveals many genes predicted to encode hydrolytic enzymes including 20 putative nucleases, some of which, we hypothesise, act to achieve this recycling. It would be expected that prey active nucleases would have secretion signals to allow their export from the *Bdellovibrio* into prey. Transcriptomic analysis of mRNA from the early stage of predation (Lambert *et al*., 2010) has highlighted two candidate-exported endonuclease-encoding genes upregulated at 30 min postintroduction of *Bdellovibrio* to prey cells; the products of which (Bd1244, Bd1934) may be involved in prey degradation. Here, we generate single and double mutants to investigate this possibility. *Bdellovibrio* have been shown to form biofilms themselves (Medina & Kadouri, 2009), and as biofilm matrices often contain many nucleic acids as an integral part of their structure (Whitchurch *et al*., 2002), we also examined the potential roles of the Bd1244 and Bd1934 nucleases in self-biofilms.

## Materials and methods

### Strains and growth media

The genome-sequenced type strain *B. bacteriovorus* HD100^T^ (Stolp & Starr, 1963; Rendulic *et al*., 2004) was used throughout this study and grown by predation on *E. coli* S17-1 (Simon *et al*., 1983) in Ca/HEPES buffer using standard culturing methods described elsewhere (Lambert *et al*., 2003). Kanamycin-resistant exconjugants containing initially single crossovers of the pK18*mobsacB* plasmid for reciprocal recombination for gene deletion were maintained on YPSC overlay plates supplemented with 50 μg mL^−1^ kanamycin sulphate with kanamycin-resistant *E. coli* S17-1 (pZMR100) prey (Rogers *et al*., 1986). Host-independent *Bdellovibrio* derivatives were isolated on PY media as described elsewhere (Shilo & Bruff, 1965; Evans *et al*., 2007).

### RT-PCR analysis of nuclease gene expression

Initial transcriptomic analysis had compared solely *Bdellovibrio* with no prey, to *Bdellovibrio* interacting with prey at 30 min after mixing (Lambert *et al*., 2010). Thus, to expand the transcriptomic ‘picture’, total RNA was extracted over the course of a semi-synchronous predatory infection and semi-quantitative RT-PCR was carried out as described previously to monitor *Bdellovibrio* nuclease gene expression (Lambert *et al*., 2006; Evans *et al*., 2007). The following primers were used: *bd1244*: Bd1244F CAATCAGTATGCGGTTCGTG Bd1244R GTTGATCACGGTGTTGTTCG, *bd1934*: Bd1934F AGCTTACGACAACCGTCTGG Bd1934R ACTGGATTTCTGCCCACTTG, *bd1431*: Bd1431F GAACGTCGAACTGCACAATG Bd1431R TAGGCATAGGCCAGGTTGTT.

### Deletion of nuclease genes in *B. bacteriovorus*

Markerless deletions of the *bd1244* and *bd1934* genes were constructed by modifications of the methods of Steyert & Pineiro (2007; Santini *et al*., 2001). One kilobase of flanking DNA from either side of the genes was amplified and joined together to give an in-frame deletion of the orfs. This was then ligated into the kanamycin-resistant suicide vector pK18*mobsacB* (Schafer *et al*., 1994) and conjugated into *B*. *bacteriovorus* HD100 [as described in (Evans *et al*., 2007)]. The resulting merodiploid exconjugants were grown with kanamycin selection on YPSC overlay plates of *E. coli* lawns before sucrose suicide selection in 5% sucrose. The double mutant was made by conjugating the *bd1934* deletion construct into a Δ*bd1244* mutant. All mutants were confirmed by sequencing, Southern blot and RT-PCR to determine that the gene had been deleted in-frame as expected and that no transcript for it was present in the mutant. As these mutants were successfully isolated using the predatory HD100 strain, there was no need to attempt to rescue them by growing host-independent strains as is necessary for genes essential for predation (Hobley *et al*., 2012).

### Nucleic acid staining during a predatory timecourse

A predatory *Bdellovibrio* prey lysate culture consisting of 10-mL Ca/HEPES buffer, 600 μL of a culture of *E. coli* S17-1 (*c*. 3 × 10^9^ cells) previously grown in YT broth for 16 h at 37 °C with shaking at 200 r.p.m.) and 200 μL of *Bdellovibrio* (*c*. 1 × 10^9^) from a previous prey lysate was incubated at 29 °C with shaking at 200 r.p.m. overnight. This was concentrated by centrifugation at 5100 ***g*** for 20 min, resuspended in 0.5-mL Ca/HEPES and added to 100 μL of a culture of *E. coli* S17-1 (grown in YT broth for 16 h at 37 °C with shaking at 200 r.p.m.) to give a semi-synchronous prey lysate with a MOI of > 3 as determined by plaque assay for the *Bdellovibrio* and colony-forming units for *E. coli*. At 15-min intervals, 9.5-μL samples was added to 0.5 μL Hoechst 33372 (a dye which stains DNA) and imaged by phase and fluorescent microscopy as described elsewhere (Altschul *et al*., 1997).

### Self-biofilm formation assay

Biofilm formation assays for host/prey independent (HI) *Bdellovibrio* were carried out as described by Medina and Kadouri (2009), but with modifications to conveniently screen many HI derivatives of *B. bacteriovorus* simultaneously. Individual HI colonies were picked into 200-μL PY media (Lambert & Sockett, 2008) in 96-well microtitre dishes and grown for 48 h at 29 °C to an average OD_600_ of 0.4–0.6. Fifty microlitres from each well were transferred into 150-μL fresh PY in PVC 96-well microtitre dishes and incubated for 48 h at 29 °C for biofilm growth. Biofilm formation in the wells was measured by washing off planktonic cells and media with sterile distilled water, staining with 200-μL 1% (w/v in ethanol) crystal violet for 15 min, destaining with 200-μL 33% (v/v) acetic acid for 15 min and transferring 150 μL of this to a separate plate to measure OD_600_. At least three biological repeats were carried out. Student's *t-*test was performed on data to test statistical significance.

### Prey biofilm depletion assay

Biofilm depletion assays for prey bacteria were carried out by modifications on the methods of Medina *et al*. (2008). *Escherichia coli* S17-1 cultures grown in LB broth at 37 °C with shaking at 200 r.p.m. for 16 h were backdiluted 1/100 in LB broth to give typical starting cell numbers of 1 × 10^7^ CFU mL^−1^, and 200 μL was added to each well in PVC 96-well microtitre dishes and incubated at 29 °C for 24 h to produce a prey biofilm. The remaining planktonic cells were washed off with Ca/HEPES buffer. Planktonic cultures of predatory *Bdellovibrio* were grown on prey until they had fully lysed (with many attack phase *Bdellovibrio* and fewer than 1 prey cell per 1000 *Bdellovibrio* visible by phase-contrast microscopy) after incubation for 16 h at 29 °C with shaking at 200 r.p.m. before being filtered through a 0.45-μm filter to remove any remaining prey cells. Two hundred microlitres (*c*. 1 × 10^9^) of these filtered predatory *Bdellovibrio* cells (in Ca/HEPES buffer) were added to the preformed *E. coli* biofilms and incubated at 29 °C for 24 h to test for predatory effects. Remaining biofilms were washed and quantified by crystal violet staining as described above. At least three biological repeats were carried out. Student's *t-*test was performed on data to test statistical significance.

## Results and discussion

### Choice of nuclease genes and transcriptional evaluation by RT-PCR

**Fig. 1:**
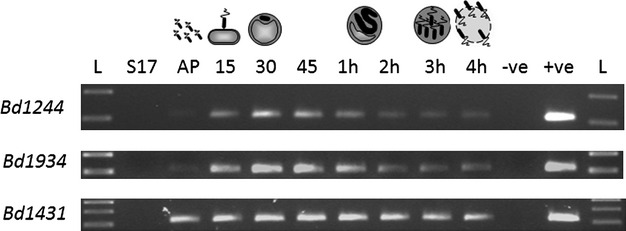
Expression patterns of genes encoding proteins with homology to exported endonucleases across the predatory cycle studied by RT-PCR. RT-PCR with transcript-specific primers on total RNA prepared from identical volumes of *Bdellovibrio bacteriovorus* HD100 predator with *Escherichia coli* S17-1 prey infection culture as the predatory infection proceeds across a timecourse. L-NEB 100-bp ladder, AP – attack phase 15–45: 15–45 min of predation, respectively, 1–4 h: 1, 2, 3 and 4 h of predation, respectively, S17: *E. coli* S17-1 only RNA as template, −ve: no template control, +ve: *B. bacteriovorus* HD100 genomic DNA as template positive control. Primers designed to predicted nuclease gene *bd1431* give a product in every sample, thus act as a positive control for the RNA, validating the lack of expression of *bd1244* and *bd1934* in the earlier and later parts of the infectious cycle. The expression peaking at 30–45 min implicates a role in interaction with the prey cells, whereas *bd1431* is constitutively expressed and hence less likely to have such a role. Above is a cartoon of the stages of predation represented by each timepoint.

There are 20 genes annotated as encoding nucleases in the *B. bacteriovorus* HD100 genome (Rendulic *et al*., 2004); these are listed in Table 1 with the top blastp hits (http://blast.ncbi.nlm.nih.gov/Blast.cgi) and smart protein domains shown (http://smart.embl-heidelberg.de/smart/set_mode.cgi). This, despite the predatory degradation of prey nucleic acids by *Bdellovibrio*, is actually not an excessive number, relative to that of nonpredatory *E. coli* MG1655 (41 annotated nucleases in a genome of 4.6 Mb). This is also the case for other predatory bacteria such as *Myxococcus xanthus* (52 annotated nucleases in a genome of 9.1 Mb). Many of the *Bdellovibrio* nuclease genes encode products with homology to ‘housekeeping’ genes such as those encoding DNA repair mechanisms (e.g. the excinucleases and others in Table 1), but many are of unknown function. Transcriptomic analysis during early bdelloplast formation, 30 min after introducing *Bdellovibrio* to prey cells, revealed that of these predicted nucleases, two endonuclease genes were upregulated at this stage: *bd1244* and *bd1934* (Lambert *et al*., 2010). RT-PCR analysis across the predatory life cycle confirmed that expression of these genes was induced upon introduction to prey cells and peaked 30–45 min after this, implicating a role for these genes in the predatory process (Figure 1), whereas in comparison, the predicted endonuclease *bd1431* was constitutively expressed and hence is more likely to be a ‘housekeeping’ gene.

**Table 1:** 

Gene	Top blastp Hit	Expect	smart	smart expect	pSORT-B
Excinucleases					
*uvrA bd0159*	Excinuclease ABC subunit A	0.0	ABC_tran	6.80e-08	Cytoplasm
*uvrC bd0254*	Excinuclease ABC subunit C	2e-70	Exonuc_X-T	2.60e-02	Cytoplasm
*uvrC bd2311*	Excinuclease ABC subunit C	1e-164	UvrC_HhH_N	6.60e-51	Cytoplasm
*uvrA bd2442*	Excinuclease ABC, A subunit	0.0	ABC_tran	1.50e-08	Cytoplasm
Exonucleases					
*xseA bd0197*	Exodeoxyribonuclease VII large subunit	1e-134	Exonuc_VII_L	2.30e-80	Cytoplasm
*xseB bd0198*	Exodeoxyribonuclease VII small subunit	1e-15	Exonuc_VII_S	5.00e-19	Cytoplasm
*bd1501*	ATP-dependent exoDNAse (exonuclease V)	6e-18	None	n/a	Unknown
*recJ bd2232*	Single-stranded DNA-specific exonuclease RecJ	1e-93	DHH domain	4.00e-07	Cytoplasm
*bd3524*	Exodeoxyribonuclease III	3e-84	Exo_endo_phos	7.10e-41	Cytoplasm
*exoA bd3670*	Exodeoxyribonuclease III	2e-87	Exo_endo_phos	2.30e-43	Cytoplasm
Endonucleases					
*bd0591*	Endonuclease III	3e-88	ENDO3c	1.70e-62	Cytoplasm
*endA bd0934*	Endonuclease I	2e-38	Endonuclease_1	3.60e-51	Extracellular
*bd1244*	Secreted nuclease	1e-106	Endonuclease_1	4.40e-51	Periplasmic
*Bd1431*	Micrococcal nuclease-like protein	3e-30	SNase (staphylococcal nuclease)	7.10e-09	Unknown
*bd1934*	Endonuclease YhcR	4e-26	SNc (staphylococcal nuclease)	7.63e-13	Unknown
*bd3507*	Endonuclease I	3e-34	Endonuclease_1	1.60e-44	Extracellular
Others					
*tatD bd1042*	Putative deoxyribonuclease	2e-73	TatD_DNase	2.50e-71	Cytoplasm
*bd3139*	UvrD/REP helicase subfamily	5e-42	UvrD-helicase	2.10e-60	Cytoplasm
*bd3140*	Double-strand break repair protein AddB	2e-08	None	n/a	Cytoplasm
*bd3695*	Type I restriction-modification system 2C	4e-53	Methylase_S	4.70e-11	Cytoplasm

**Fig. 2:**
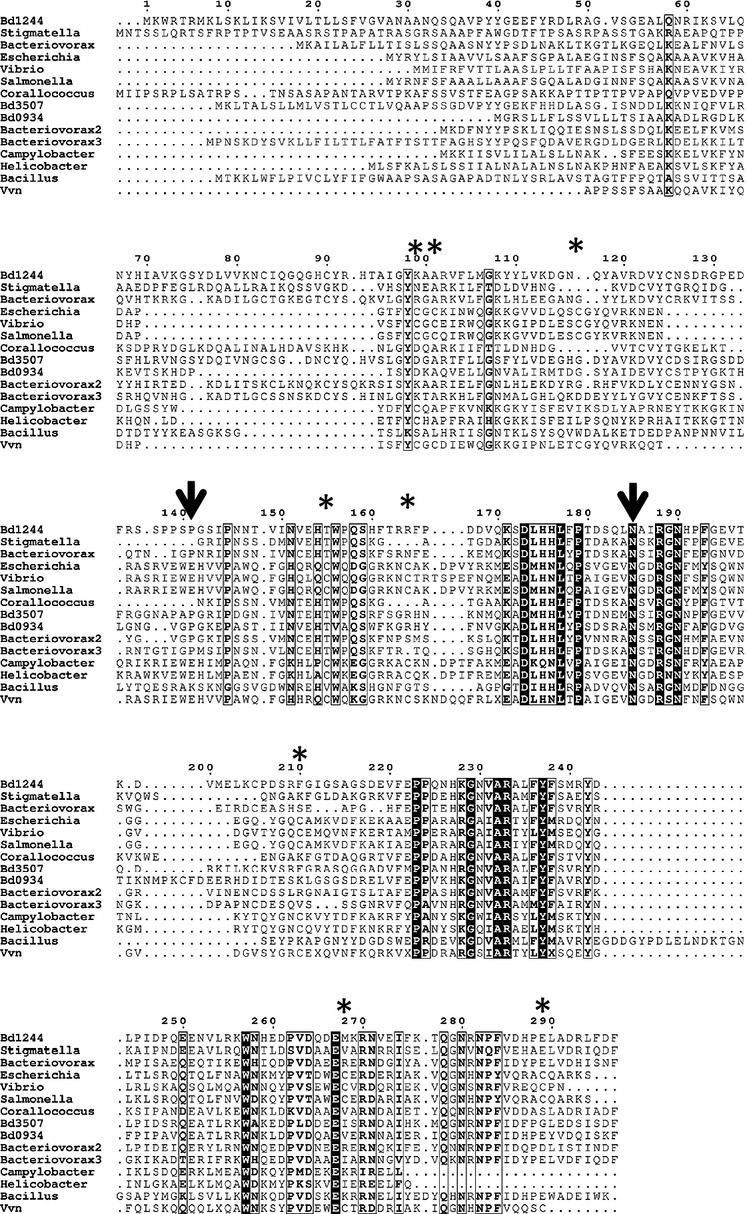
Multiple sequence alignment of predicted endonuclease I proteins created with the ClustalW program. The metal-binding residues known in the *Vibrio vulnificus* protein are indicated by arrows and are conserved amongst the *Gamma*- and *Epsilonproteobacteria*, but only one (Asn127-*Vibrio* numbering) is conserved, whilst the other (Glu79) is not amongst the *Delta proteobacteria*, suggesting that they have a different structure. Asterisks are above the cysteine residues that form disulphide bridges in the *Vibrio* protein are also conserved in the *Gamma*- and *Epsilonproteobacteria* and not the *Deltaproteobacteria*. Bd number refer to the predicted *Bdellovibrio* proteins in Table 1, *Bacteriovorax* sequences are predicted proteins from *Bacteriovorax marinus* (Crossman *et al*., 2012), Vvn is the sequence of the *V. vulnificus* protein (Seper *et al*., 2011) and the others are from genera as named.

**Fig. 3:**
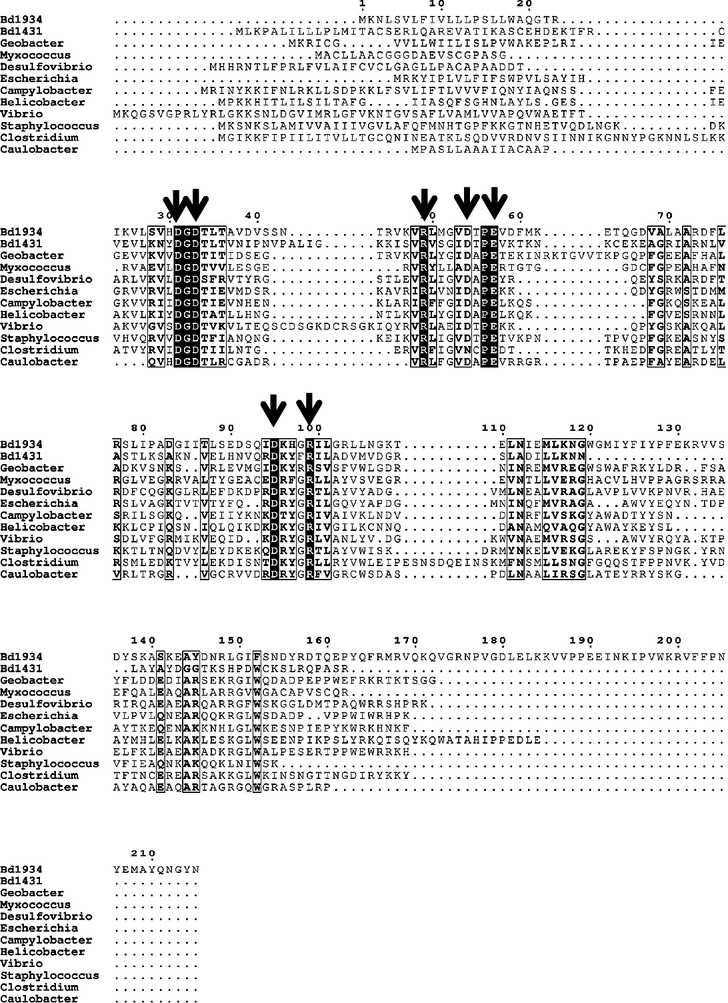
Multiple sequence alignment of predicted SNase proteins created with the ClustalW program. Arrows show all of the conserved residues known to be involved in metal or substrate binding in *Staphylococcus aureus,* which are completely conserved in the *Deltaproteobacteria*, suggesting that these are likely acting in the same way. Bd numbers refer to the predicted *Bdellovibrio* proteins in Table 1, and the others are from genera as named.

Analysis of the sequences using the SignalP program indicated that *bd1244* and *bd1934* products (and also *bd0934*, *bd1431* and *bd3507*) were likely to have a signal sequence for sec-dependent transport across the inner membrane and are therefore predicted to be at least extracytoplasmic (and possibly exported beyond out of the cell by other transport systems such as PulD), and therefore, we considered them unlikely to be housekeeping genes for chromosome maintenance. Table 1 shows that the five endonucleases with predicted signal sequences have conserved domains placing them in the endonuclease I superfamily (the staphylococcal nuclease is part of this family), the biological functions of which are unclear in most other bacteria. In pathogenic bacteria such as *Streptococcus pneumoniae*, an extracellular nuclease of this family degrades DNA meshes of neutrophil extracellular traps to escape host immune responses (Altschul *et al*., 1997), whilst conversely, in *Vibrio cholerae*, extracellular nucleases of this family are involved in biofilm formation (Seper *et al*., 2011), suggesting a diversity of functions for these. Figure 2 shows a multiple sequence alignment of different predicted endonuclease I protein sequences using the ClustalW program. This shows that the two metal-binding residues known in the *Vibrio vulnificus* protein (Li *et al*., 2003) are conserved amongst the gamma and epsilon proteobacteria, but only one (Asn127-*Vibrio* numbering, arrow on Figure 2) is conserved, whilst the other (Glu79) is not amongst the delta proteobacteria, suggesting that they have a different structure. Similarly, the cysteine residues that form disulphide bridges in the *Vibrio* protein (Li *et al*., 2003; below the asterisks in Figure 2) are also conserved in the gamma and epsilon proteobacteria and not the delta proteobacteria. Figure 3 shows a multiple sequence alignment of different predicted SNase protein sequences and in contrast to the endonuclease I alignment shows that all of the conserved residues known to be involved in metal or substrate binding in *Staphylococcus aureus* (Ponting, 1997) are completely conserved in the delta proteobacteria, suggesting that these are likely a similar structure and mode of action.

### Nuclease deletion mutants

The *B. bacteriovorus* HD100 genome has many copies of genes with similar functions (Rendulic *et al*., 2004) and often display redundancy with single mutants sometimes having no obvious lack of function (Lambert *et al*., 2006; Morehouse *et al*., 2011). Therefore, to investigate the roles of the two nucleases implicated in prey interaction, in addition to generating deletion mutations of *bd1244* and *bd1934*, we also generated a double Δ*bd1244bd1934* mutant with both genes deleted. The genes were deleted such that only the first two and last three codons remained, so that the deletion was in-frame and therefore unlikely to affect surrounding genes. Deletion was confirmed by sequencing the regions in the mutants, Southern blot and RT-PCR to confirm the absence of transcript (data not shown). The mutants showed no obvious morphological differences and could prey upon *E. coli* in a manner apparently identical to wild type, with semi-synchronous, planktonic, predatory cultures (MOI > 3) showing *Bdellovibrio* swimming rapidly, attaching to and entering, then growing within and lysing prey cells within 3–5 h as did the wild-type HD100 strain. A predation assay using luminescent prey was performed as described previously (Lambert *et al*., 2003) and showed no difference in rate of predation between the mutants and wild type (data not shown).

### Hoechst 33372 staining of nucleoid material throughout the predation process

To observe any differences in prey DNA degradation, semi-synchronous cultures of wild-type and double Δ*bd1244bd1934* mutant were set up (MOI > 3) on *E. coli* prey, and samples were taken and stained with Hoechst 33372, which stains DNA and can be observed under fluorescence and phase-contrast microscopy. The *Bdellovibrio* cells showed a very intense signal as their large 3.8 Mb genome is compacted into a small space, and the *E. coli* prey had a more diffuse signal reflecting their less concentrated DNA. No obvious differences in the disappearance of the prey DNA fluorescence upon predation by the different strains were observed. It may be that this assay was not sensitive enough to monitor minor differences in the rate of DNA breakdown or that the nucleases have an altogether different role in the *Bdellovibrio* lifestyle, which is induced upon signals from detecting the presence of prey, such as biofilm formation. We tested this idea further for the single and double nuclease mutants.

### Biofilm formation by HI *Bdellovibrio*

**Fig. 4:**
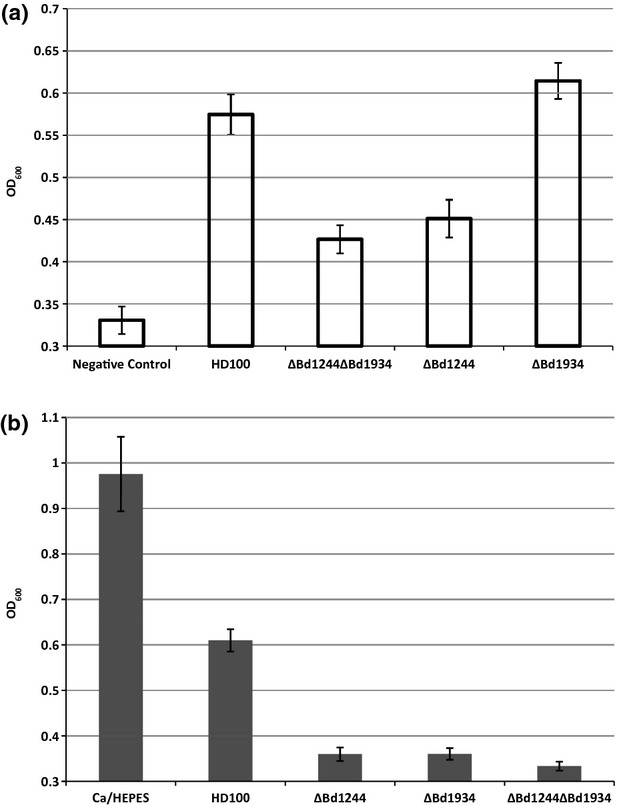
(a) Self-biofilm formation (as crystal violet staining intensity) by *Bdellovibrio* HI isolates. HI isolates from each of the nuclease-mutant and wild-type *Bdellovibrio bacteriovorus* HD100 were incubated in PVC microtitre plates in PY media for 48 h. The resulting biofilms were washed and stained with crystal violet. The stain was then removed and quantified by OD_600_ readings. The *bd1244* mutant and double mutant formed less self-biofilm than the wild-type and *bd1934* mutant. (b) Elimination of preformed *Escherichia coli* prey biofilms by *Bdellovibrio* HD strains. *Escherichia coli* biofilms were pregrown in PVC microtitre plates for 24 h, and then, filtered cultures of *Bdellovibrio* (log8 ± log0.5 PFU) were added and incubated for a further 24 h. The remaining prey biofilms were washed and stained with crystal violet. The stain was then removed and quantified by OD_600_ readings. The nuclease-mutant *Bdellovibrio* strains surprisingly clear the biofilm more efficiently than the wild type. Ca/HEPES buffer was added in place of *Bdellovibrio* as a negative control.

Medina and Kadouri (2009) describe the ability of HI *Bdellovibrio* to form biofilms and it is known that extracellular DNA (eDNA) is often important in biofilm formation of other bacteria (Whitchurch *et al*., 2002), so we tested the ability of the nuclease mutants to form biofilms. As HI derivatives of *Bdellovibrio* have, without additional deliberately introduced mutations, widely varying phenotypes of morphology, growth rate and cell coloration (Seidler & Starr, 1969; Barel & Jurkevitch, 2001); many different HI derivatives of each mutant were tested in these experiments by growth in PVC microtitre plates in PY for 48 h by the method of Medina and Kadouri (2009). This was to ensure that differences between strains were not merely a result of normal variation of HI derivatives. Figure 4a shows that both the Δ*bd1244* mutant and the double Δ*bd1244bd1934* mutant formed *Bdellovibrio* HI biofilms less efficiently than wild-type and the Δ*bd1934* mutant. These differences were significant as determined by Student's *t*-test (*P* > 0.001), implying that the Bd1244 nuclease is involved in biofilm formation. Extracellular nucleases of this endonuclease I family have been shown to alter biofilm formation in *Vibrio cholerae* (Seper *et al*., 2011) in that instance deletion of the two nuclease genes, both individually and together, resulted in greater biofilm production with the nucleases implicated in mechanisms of biofilm architecture, dispersal and nutrient acquisition. This disparity could be a result of different growth media used as this is known to have a profound effect on biofilm formation (Bininda-Emonds, 2005). As HI *Bdellovibrio* have a stringent requirement for nutrient-rich peptone–yeast extract-based (PY) media, this media effect could not be tested further. Different species of bacteria produce widely varying amounts of eDNA in biofilms (Steinberger & Holden, 2005) with a diversity of roles, so it is easily conceivable that the *Bdellovibrio* nucleases have different roles to that in *Vibrio* In diverse bacteria, eDNA has been shown to have different roles, for example, DNaseI detached biofilms of *Staphylococcus aureus,* but not *Staphylococcus epidermidis*; in *Caulobacter*, eDNA of biofilms binds to the holdfast of stalked cells and inhibits cell attachment to the biofilm (Berne *et al*., 2010). *Bdellovibrio* also have specialised attachment processes (to prey) allowing them to distinguish between prey and nonprey cells [although the details of this are elusive (Varon & Shilo, 1969) and may not be the same as for *Caulobacter*]. Therefore, it is possible that an analogous process is happening here, and in the absence of nucleases, a build-up of eDNA inhibits further planktonic cells from joining the biofilm that results in slower biofilm formation by the Bd1244 mutant.

Several attempts to clone the *Bdellovibrio* nuclease genes to complement the mutants were unsuccessful. This is likely due to the genes being lethal expressed *in trans* in *E. coli*. As the cysteine residues that form disulphide bridges in endonuclease I (Li *et al*., 2003) are not conserved in Bd1244 of *Bdellovbrio*, it is possible that at least some of the protein can fold into an active conformation before export and this could result in lethal degradation of the cloning strain genome.

### Breakdown of preformed prey biofilms by *Bdellovibrio*

*Bdellovibrio* have been shown to eliminate preformed biofilms of *E. coli* (Kadouri & O'Toole, 2005). To investigate any possible role of nucleases in this, we tested the mutants using the methods of Medina *et al*. (2008) by pregrowing a biofilm of *E. coli* on PVC microtitre plates for 24 h, washing and then applying filtered *Bdellovibrio* for 24 h. Figure 4b shows that surprisingly, all of the mutant strains eliminated preformed biofilms more efficiently than wild-type strain HD100. These differences were significant as determined by Student's *t*-test (*P* ≫ 0.001). Plaque enumerations confirmed that there were similar numbers (log8 ± log0.5) of viable *Bdellovibrio* in the samples initially added.

One interpretation of this interesting result is that for each of the nuclease mutants, reduced modification of the eDNA in the prey biofilm partially restricts *Bdellovibrio* escape from the biofilm matrix. This could then cause increased localised predation and hence more prey death in the biofilm compared to wild-type *Bdellovibrio* predation.

The disparity between the Δ*bd1934* mutation causing an effect here, but not in the HI self-biofilms, may be due to different *Bdellovibrio* gene expression caused by the different media conditions. Alternatively, it may be that the disruption of *bd1934* has resulted in overproduction of another nuclease in that strain, which breaks down biofilm nucleic acids. RT-PCR with primers annealing to *bd1244*, *bd1934* and *bd1431* (as a sample of putatively exported endonucleases that may have compensated in the other two mutants) showed no evidence of this in any of the mutants (data not shown), but there is a possibility that abnormal expression of other nucleases may be overcompensating for the mutant defect. This counter-intuitive result, of increased predation in nuclease-defective mutants, is interesting to note should *Bdellovibrio* ever fulfil its potential as a biocontrol agent. It may be possible to engineer strains that are more potent than wild-type strains at eradicating prey in specific situations such as biofilms.
